# Body esteem and sexual life in Turkish patients with cancer

**DOI:** 10.1007/s00520-026-10903-8

**Published:** 2026-06-26

**Authors:** Rukiye Burucu, Tuba Korkmaz Aslan

**Affiliations:** 1 Department of Internal Medicine Nursing, Seydişehir Kamil Akkanat Faculty of Health Sciences, Necmettin Erbakan University, Seydişehir, Konya Turkey; 2 Department of Mental Health and Diseases Nursing, Seydişehir Kamil Akkanat Faculty of Health Sciences, Necmettin Erbakan University, Seydişehir, Konya Turkey

**Keywords:** Cancer, Neoplasms, Body esteem, Sexual health, Oncology nursing, Supportive care

## Abstract

**Purpose:**

This study aimed to determine the factors affecting body esteem and sexual life among Turkish individuals diagnosed with cancer, exploring the relationship between treatment-related physical changes and psychosexual well-being.

**Methods:**

A descriptive and correlational design was employed. The sample consisted of 103 patients receiving treatment in an oncology unit of a medical faculty. Data were collected between November 2022 and January 2023 using a personal information form, the Body-Esteem Scale (BES), and the Arizona Sexual Experiences Scale (ASEX).

**Results:**

The mean age of participants was 54.72 ± 9.39 years. Findings showed that body self-esteem scores were below average (39.06 ± 11.97) and mean ASEX scores (18.34 ± 6.37) were well above the clinical threshold of 11. Multiple regression analysis showed that there were variables predicting body self-esteem (*R*^2^ = 0.199, *p* < 0.001). Sexual life was significantly affected by gender, BMI, and cancer stage, and women reported higher levels of dysfunction (*p* < 0.001). A significant negative correlation was found between the BES “attribution” subscale and total ASEX scores (*r* = −0.284, *p* = 0.004).

**Conclusion:**

Cancer treatment and its complications are significant determinants of both body image and sexual health in Turkish patients. This study shows that clinical factors such as cancer-related weight loss, edema, and catheter presence significantly affect body self-esteem, while sexual dysfunction is primarily influenced by gender, BMI, and disease stage. Addressing these psychosexual challenges is essential for improving the overall quality of life for individuals progressing through the cancer process.

## Introduction

Cancer is a common disease and one of the leading causes of death [1]. People with cancer experience many problems, including distorted body image [2, 3] as well as aesthetic problems [4], and sexual problems [5]. These problems harm their body esteem [6].

Body esteem is one’s evaluation of one’s body [7]. People with high body esteem know their bodily desires [8]. People with high body esteem are likely to have better sex lives. Sexuality is essential for both sexes [9]. In addition to physical changes, cultural norms significantly shape how patients perceive and communicate their sexual needs. In Turkish society, sexual health remains a sensitive and often-neglected topic due to traditional norms, religious beliefs, and the profound concept of “mahremiyet” (extreme privacy). Within this cultural framework, sexuality is not merely a biological act but a confidential taboo associated with shame and sin, which is often not discussed even within the family [10]. Particularly when battling a traumatic and life-threatening illness like cancer, expressing sexual needs may be perceived by society, and even by healthcare professionals, as a “secondary concern” or an inappropriate issue relative to the gravity of the disease. As noted by Korukcu et al. (2017), this cultural pressure and sense of shame make it difficult for patients to report disruptions in their body image or sexual problems to healthcare providers. This environment leads to sexual dysfunctions remaining hidden in clinical settings and contributes to “social desirability bias” in research, potentially causing the data to represent the situation as less severe than it truly is [11]. When faced with a life-threatening illness like cancer, patients may prioritize survival over sexual well-being, leading to a “silence” in clinical settings [12]. There is a positive correlation between an active sex life and body image. Therefore, providing structured sexual counseling and supportive care to patients undergoing cancer treatment will be beneficial [9, 13]. The adequacy of body esteem also positively affects the quality of sexual life [14].

Sexual health is not only the absence of sexual dysfunction and disability but also involves physical, emotional, mental, and social well-being, a positive and respectful approach to sexuality and sexual relations, and the possibility of having pleasurable and safe sexual experiences free from coercion, discrimination, and violence [15]. One’s sexual health depends on how one perceives one’s body [16]. People should identify their sexual problems [17] because society attributes specific roles to its members within the context of their sexuality and pressures them to conform to those roles [18]. Turkish society also has certain sexual norms. Therefore, Turkish people have difficulty discussing their sexual problems [10]. Furthermore, individuals diagnosed with severe medical conditions, such as cancer, may deprioritize both their sexuality and the perception of their sexual dysfunctions as primary concerns [12]. People with cancer tend to put their sex lives on the back burner when diagnosed with cancer [19]. Therefore, some researchers recommend that nurses provide sexual counseling services to patients with cancer [20].

Sulamış argues that body esteem and sexual life affect each other [16]. However, no researchers have examined the relationship between body esteem and sexual life in Turkish patients with cancer. Therefore, this paper investigated what factors affected the body esteem and sex lives of Turkish patients with cancer. We believe our results will raise the awareness of healthcare professionals and encourage multidisciplinary teams to provide holistic care to their patients with cancer.

*This study was guided by four primary research questions*: first, to identify what kind of body esteem Turkish patients with cancer have; second, to describe how the sex lives of Turkish patients with cancer are; third, to evaluate if body esteem affects the sex lives of Turkish patients with cancer; and finally, to determine if there is a correlation between body esteem and sexual life.

## Material and methods

This was a descriptive and correlational study.

Population: The study population consisted of all patients with cancer followed up or treated in the oncology unit of a university hospital in Türkiye.

Sample: A power analysis (G*Power 3.1.9.4) was performed to determine the sample size based on Tarkowska et al., who focused on sexual dysfunctions among patients with and without cancer [21]. The results showed that a sample of 88 would be large enough to detect significant differences (0.05 error, 0.85 power, and 0.579 effect size). We recruited 103 patients to avoid missing data. The study had a post hoc power of 0.898.

*The inclusion criteria* for the study were being 18 years of age or older, being sexually active (having engaged in at least one physical sexual encounter within the last 6 months), volunteering to participate in the research, and receiving active chemotherapy (defined as having completed at least one cycle of chemotherapy and currently continuing the treatment protocol) for a non-genital malignancy. The sample primarily consisted of patients with digestive system cancers (57.3%) and other non-genital malignancies; a detailed distribution of these cancer sites and diagnostic groups is provided in Table 1. *The exclusion criteria* included having communication or cognitive impairments that would hinder the data collection process and having a medical history of chronic sexual dysfunction diagnosed prior to the cancer diagnosis. Furthermore, patients with male and female reproductive system cancers (e.g., prostate, testicular, ovarian, endometrial, or cervical cancers) were excluded, as these malignancies and their associated surgical or radiotherapy procedures carry a high risk of causing direct anatomical and mechanical damage to the sexual organs, which could potentially confound the study results.

**Table 1:** Sociodemographic characteristics (*n* = 103)

Sociodemographic characteristics	Min–max	Mean	SD
Age (year)	24–65	54.720	9.397
Number of children	0–11	2.83	1.815
Time to diagnosis	1–9	3.18	2.195
BMI	15.23–40.69	25.004	5.139
BES total	13–68	39.068	11.970
BES appearance	5–33	17.475	5.254
BES body weight	2–22	13.660	5.170
BES attribution	1–20	7.932	4.078
ASEX total	6–30	18.349	6.371
		***N***	**%**
Gender	Woman	51	49.5
	Man	52	50.5
Marital status	Married	97	94.2
	Single	6	5.8
Education (degree)	Illiterate	4	3.9
	Primary school	57	55.3
	High school	28	27.2
	Bachelor’s	14	13.6
Income	Income < expense	40	38.8
	Income = expense	51	49.5
	Income > expense	12	11.7
Place of residence	City	65	63.1
	District	20	19.4
	Village	18	17.5
Tobacco use	Yes	16	15.5
	No	69	67.0
	Quit	18	17.5
Alcohol use	Yes	5	4.9
	No	90	87.4
	Quit	8	7.8
Diagnosis	Respiratory system cancer	9	8.7
	Digestive system cancer	59	57.3
	Others	35	34.0
Medical diagnosis	Digestive system cancer	59	53.7
	Respiratory system cancer	9	8.7
	Other	35	34.0
Cancer stage	1^a^	14	13.6
	2^b^	36	35.0
	3^c^	23	22.3
	4^d^	30	29.1
Do you have comorbidities?	Yes	41	39.8
	No	62	60.2
Does your spouse look down on you?	Yes	11	10.7
	No	92	89.3
Does your spouse support you?	Yes	89	86.4
	No	14	13.6
Are you scared of being abandoned by your spouse?	Yes	7	6.8
	No	96	93.2
Have you been informed about bodily changes due to cancer?	Yes	83	80.6
	No	20	19.4
Have you put on weight?	Yes	21	20.4
	No	82	79.6
Have you lost weight?	Yes	58	56.3
	No	45	43.7
Do you have edema?	Yes	8	7.8
	No	95	92.2
Is your complexion yellow-gray?	Yes	31	30.1
	No	72	69.9
Has your hair gotten thinner?	Yes	33	32.0
	No	70	68.0
Do you have catheters?	Yes	10	9.7
	No	93	90.3
Are your bodily changes noticeable/visible?	Yes	64	62.1
	No	39	37.9

### Data collection

The research data were collected face-to-face using a personal information form, the Body-Esteem Scale (BES), and the Arizona Sexual Experiences Scale (ASEX). In a cultural context where sexuality is considered a sensitive and often taboo subject, the face-to-face interview method was intentionally preferred over self-report surveys. This approach allowed the researcher to establish a trusting rapport with the participants, provide immediate clarifications to ensure the accurate understanding of the ASEX items, and minimize data loss by addressing participants’ hesitations regarding sensitive topics. Furthermore, this method was adopted to prevent the physical fatigue and concentration difficulties often experienced by patients during active chemotherapy cycles from hindering the data collection process, ensuring that the data were gathered in a supportive clinical environment while strictly adhering to confidentiality principles.

The selection of specific clinical variables for the regression model, such as visible catheter use, edema, and weight loss, was strategically aligned with the clinical profile of the study population. Since more than half of the sample (57.3%) consisted of patients with gastrointestinal system cancers, these variables represent the most common and distressing physical complications of their illness and treatment trajectory. In this patient group, long-term external catheters (e.g., ports or central lines) are routinely used for chemotherapy and parenteral nutrition. Additionally, symptoms such as cancer cachexia or ascites-related edema significantly alter body symmetry and physical appearance. Thus, these variables were selected as primary clinical markers known to impact the “Appearance” and “Attribution” dimensions of body image, as well as the physical comfort necessary for sexual well-being.

### Personal information form

The personal information form was based on a literature review [2, 3, 5]. The form consisted of 21 items on age, gender, income, education, comorbidity, tobacco and alcohol use, cancer stage, etc.

### Body-esteem scale

The Body-Esteem Scale (BES) was developed by Mendelson, Mendelson, and White (2001) and adapted to Turkish by Sevi-Tok and Gedik (2019). It is a self-report measure for assessing respondents’ feelings about their own bodies and appearance. The instrument consists of 23 items rated on a 5-point Likert-type scale (0 = never to 4 = always). The instrument has three subscales: appearance (Items 1, 6, 7, 9, 11, 13, 15, 17, 21, and 23), body weight (Items 3, 4, 8, 10, 16, 18, 19, and 22), and attribution (Items 2, 5, 12, 14, and 20). Nine items are reverse-scored (Items 4, 7, 9, 11, 13, 17, 18, 19, and 21). The total score ranges from 0 to 92. The instrument has no cut-off point. Higher scores indicate higher body esteem. The Turkish version has a Cronbach’s alpha of 0.94, while the subscales “appearance,” “body weight,” and “attribution” have Cronbach’s alpha values of 0.91, 0.93, and 0.79, respectively [22]. In the present study, the scale had a Cronbach’s alpha of 0.764.

### Arizona sexual experiences scale

The Arizona Sexual Experiences Scale (ASEX) was developed by McGahuey et al. (2000). The instrument assesses sexual functioning, excluding sexual orientation and relationships with sexual partners. It consists of five items rated on a scale of 1 to 6. The total score ranges from 5 to 30, with higher scores indicating the presence of sexual dysfunction. A total score of 19 or more, a score of 5 or 6 on any item, or a score of 4 on three or more items indicates sexual dysfunction [23]. However, Soykan reported a scale score of ≥ 11 as the cut-off point for sexual dysfunction. The scale was adapted to Turkish by Soykan, who reported a Cronbach’s alpha of 0.90 [24]. In the present study, the scale demonstrated a Cronbach’s alpha of 0.933, and the cut-off score for sexual dysfunction was set at ≥ 11 based on the Turkish validation study by Soykan (2004). Scores equal to or exceeding this threshold indicate the presence of significant sexual dysfunction.

#### Data collection

The data were collected face-to-face between November 2022 and January 2023. Data collection took an average of 15 min. During the interviews, the researchers took protective measures to prevent participants from being exposed to unfavorable situations (infection transmission, etc.). The researchers ensured privacy and confidentiality. The interviews were completed in an interview room where only the researcher and the participant were present.

In this study, sexual function (ASEX total score) was defined as the primary *dependent* variable for the regression analysis. Body esteem (BES total score and subscales) was treated as the primary *independent* (predictor) variable to determine its impact on sexual life. Other sociodemographic and clinical characteristics (e.g., age, gender, BMI, cancer stage) were also analyzed as independent variables to evaluate their influence on both body esteem and sexual health.

### Ethical considerations

The study was approved by the Necmettin Erbakan University Health Sciences Scientific Research Ethics Committee (2022/295: ID: 11 483). Permission was obtained from the Necmettin Erbakan University Faculty of Medicine Hospital (E-14567952-900-259821). Informed consent was obtained from all participants. The research was conducted according to the ethical principles of the World Medical Association’s Declaration of Helsinki. The study adhered to the Strengthening the Reporting of Observational Studies in Epidemiology (STROBE) guidelines.

### Data analysis

The data were analyzed using the Statistical Package for the Social Sciences (IBM SPSS, 25.0) at a significance level of 0.05. Normality was tested based on skewness and kurtosis values between −1 and +1 [25]. The results showed that the data were normally distributed. Percentages, mean, standard deviation, and minimum and maximum values were used for descriptive data. The data were analyzed using independent samples *t*-test and one-way ANOVA. Fisher’s least significant difference (LSD) test was used for post hoc pairwise comparisons to determine the source of difference. The relationship between scale scores was determined using the Pearson correlation coefficient.

To determine the extent to which body esteem and relevant sociodemographic/clinical factors predict sexual dysfunction, multiple linear regression analysis was conducted. The variables included in the regression model (e.g., catheter use) were selected based on both clinical observations and existing literature, which indicates their significant impact on body image and sexual quality of life [26–28]. Considering that the majority of the sample (57.3%) consisted of patients with digestive system cancers, the presence of medical devices such as catheters, drains, or stomas was evaluated as a primary factor disrupting the patient’s sexual self-concept and physical comfort. These variables were incorporated into the model to determine the predictive effects of physical changes experienced during the active treatment process on psychosexual outcomes.

## Results

The sociodemographic and clinical characteristics of the 103 participants are summarized in Table 1. The study population had a mean age of 54.72 ± 9.39 years and a mean BMI of 25.00 ± 5.14. Participants had more than two children on average (2.83 ± 1.81) and a mean time since diagnosis of 3.18 ± 2.19 years. The mean total score for the Body-Esteem Scale (BES) was 39.06 ± 11.97, indicating a relatively low level of body esteem among the participants. Furthermore, the mean Arizona Sexual Experiences Scale (ASEX) score was 18.34 ± 6.37, which is substantially higher than the established cut-off point of 11 for the Turkish population. This finding demonstrates that the majority of the patients in the sample experienced clinically significant levels of sexual dysfunction. Collectively, these results suggest that patients in the sample experience significant challenges regarding both their body image and sexual functioning (Table 1).

Table 2 presents the comparison of BES and ASEX scores according to the sociodemographic and clinical characteristics of the participants. Regarding body esteem (BES), participants who did not experience cancer-related weight loss had a significantly higher mean BES total score (41.91 ± 11.85) compared to those who did. Participants without edema (39.85 ± 11.82) and those without catheters (39.94 ± 11.67) had significantly higher body esteem scores than those experiencing these conditions (*p* < 0.05). Analysis of the “Appearance” subscale revealed that participants who were not looked down on by their spouses (16.94 ± 5.00) and those who did not fear abandonment by their spouses (17.21 ± 5.24) had significantly higher scores than their counterparts (*p* < 0.05). Additionally, participants without edema had significantly higher appearance scores (17.89 ± 5.13) than those with edema (12.50 ± 4.17, *p* = 0.005). In the “Body Weight” subscale, significantly higher scores were found among participants who were informed about bodily changes, those without catheters, and those who smoked (*p* < 0.05). In the “Attribution” subscale, male participants (8.69 ± 3.77) scored significantly higher than female participants (7.15 ± 4.26, *p* = 0.050). Regarding sexual life (ASEX) findings, female participants had a significantly higher mean ASEX score (20.58 ± 6.03) than males (16.15 \pm 5.96), indicating a higher level of sexual dysfunction among women (*p* = 0.000). Non-smokers (19.46 ± 5.95) and those who had quit smoking (18.16 ± 7.66) had higher mean ASEX scores compared to active smokers (13.75 ± 4.50, *p* = 0.004). Cancer stage significantly influenced sexual function; participants with stage I cancer had significantly lower mean ASEX scores (indicating better sexual function) compared to those with stages II, III, and IV (*p* = 0.019). Overall, the participants’ mean BES score remained below average, while the mean ASEX score exceeded the established cut-off point of 11 (Table 2).

**Table 2:** Scale scores (*n *= 103)

Variables		BES total		BES appearance		BES body weight		BES attribution		ASEX total	
		Mean	SD	Mean	SD	Mean	SD	Mean	SD	Mean	SD
Gender	Woman	37.843	12.581	17.333	5.562	13.352	5.480	7.156	4.267	20.588	6.037
	Man	40.269	11.332	17.615	4.982	13.961	4.882	8.692	3.770	16.153	5.961
		*t* = 2.767 *p*: 0.306		*t* = 0.537 *p*: 0.787		*t* = 3.499 *p*: 0.553		*t* = 1.073 ***p*: 0.0500**		*t* = 0.146 ***p*: 0.000**	
Marital status	Married	39.31	11.826	17.567	5.269	13.752	5.133	8.000	4.059	18.185	6.297
	Single	35.000	14.724	16.000	5.215	12.166	6.047	6.833	4.622	21.000	7.615
		*t* = 0.706 *p*: 0.394		*t* = 0.30 *p*: 0.481		*t* = 0.007 *p*: 0.469		*t* = 0.049 *p*: 0.499		*t* = 0.090 *p*: 0.296	
Does your spouse support you?	Yes	38.853	11.849	17.179	4.985	13.674	5.359	8.000	4.139	17.966	6.240
	No	40.428	13.095	19.357	6.628	13.571	3.916	7.500	3.777	20.785	6.896
		*t* = 0.111 *p*: 0.650		*t* = 1.327 *p*: 0.150		*t* = 3.322 *p*: 0.945		*t* = 0.805 *p*: 0.672		*t* = 0.078 *p*: 0.124	
Does your spouse look down on you?	Yes	44.00	8.9554	21.909	5.430	14.636	3.107	7.454	2.696	20.181	8.874
	No	38.478	12.186	16.945	5.004	13.543	5.364	7.989	4.220	18.130	6.033
		*t* = 1.586 *p*: 0.149		*t* = 2.245 ***p*: 0.003**		*t* = 4.836 *p*: 0.510		*t* = 4.197 *p*: 0.683		*t* = 6.873 *p*: 0.315	
Are you scared of being abandoned by your spouse?	Yes	44.857	9.388	21.000	4.320	15.857	4.298	8.000	2.768	23.285	7.653
	No	38.645	12.068	17.218	5.241	13.500	5.211	7.927	4.168	17.989	6.161
		*t* = 549 *p*: 0.186		*t* =.266 ***p*: 0.006**		*t* = 0.606 *p*: 0.246		*t* = 3.563 *p*: 0.964		*t* = 0.745 *p*: 0.119	
Have you been informed about bodily changes due to cancer?	Yes	40.144	11.470	17.674	4.752	14.192	5.166	8.277	4.112	18.361	6.554
	No	34.600	13.244	16.650	7.058	11.450	4.684	6.500	3.692	18.300	5.704
		*t* = 2.147 *p*: 0.063		*t* = 5.997 *p*: 0.436		*t* = 424 ***p*: 0.033**		*U* = 0.059 *p*: 0.080		*t* = 0.891 *p*: 0.969	
Have you put on weight?	Yes	36.333	13.712	16.238	6.378	12.142	6.334	7.952	3.787	18.428	7.145
	No	39.768	11.471	17.792	4.920	14.048	4.796	7.926	4.171	18.329	6.206
		*t* = 1.734 *p*: 0.243		*t* = 1.153 *p*: 0.228		*t* = 4.690 *p*: 0.132		*t* = 0.067 *p*: 0.980		*t* = 1.057 *p*: 0.950	
Have you lost weight?	Yes	36.862	11.685	16.724	5.018	13.069	5.173	7.069	3.995	18.724	6.251
	No	41.911	11.858	18.444	5.446	14.422	5.123	9.044	3.954	17.866	6.562
		*t* = 0.108 ***p*: 0.033**		*t* = 0.012 *p*: 0.100		*t* = 0.063 *p*: 0.189		*t* = 0.001 ***p*: 0.014**		*t* = 0.119 *p*: 0.501	
Do you have edema?	Yes	29.750	10.194	12.500	4.174	12.000	5.014	5.250	3.845	21.750	6.273
	No	39.852	11.821	17.894	5.135	13.800	5.185	8.157	4.035	18.063	6.329
		*t* = 0.118 ***p*: 0.021**		*t* = 0.180 ***p*: 0.005**		*t* = 0.016 *p*: 0.347		*t* = 0.060 *p*: 0.052		*t* = 0.104 *p*: 0.116	
Is your complexion yellow-gray?	Yes	36.387	11.511	17.387	6.124	12.451	4.717	6.548	3.622	19.129	7.477
	No	40.222	12.057	17.513	4.878	14.180	5.300	8.527	4.141	18.013	5.858
		*t* = 0.111 *p*: 0.137		*t* = 4.690 *p*: 0.911		*t* = 0.601 *p*: 0.120		*t* = 0.583 ***p*: 0.023**		*t* = 4.741 *p*: 0.418	
Has your hair gotten thinner?	Yes	37.363	12.886	16.57	5.777	12.818	5.162	7.969	4.065	17.484	5.783
	No	39.871	11.523	17.900	4.975	14.057	5.163	7.914	4.113	18.757	6.632
		*t* = 0.161 *p*: 0.324		*t* = 0.267 *p*: 0.234		*t* = 0.60 *p*: 0.258		*t* = 159 *p*: 0.949		*t* = 2.429 *p*: 0.347	
Do you have catheters?	Yes	30.900	12.151	15.600	5.699	9.500	4.527	5.800	3.765	21.100	4.909
	No	39.946	11.679	17.677	5.196	14.107	5.055	8.161	4.062	18.053	6.461
		*t* = 0.036 ***p*: 0.022**		*t* = 0.602 *p*: 0.237		*t* = 0.277 ***p*: 0.007**		*t* = 0.187 *p*: 0.082		*t* = 2.671 *p*: 0.152	
Are your bodily changes noticed/visible?	Yes	37.531	11.779	17.234	5.644	13.093	4.875	7.203	3.916	19.500	6.556
	No	41.589	12.003	17.871	4.583	14.589	5.561	9.128	4.105	16.461	5.642
		*t* = 0.036 *p*: 0.095		*t* = 1.808 *p*: 0.553		*t* = 986 *p*: 0.155		*t* = 0.114 ***p*: 0.019**		*t* = 1.981 ***p*: 0.018**	
Alcohol use	Yes	49.600	12.641	21.800	3.962	15.500	5.451	11.400	5.813	15.600	6.985
	No	38.888	11.747	17.411	5.214	16.400	4.098	7.811	4.029	18.666	6.354
	Quit	34.500	11.771	15.500	5.451	11.875	4.911	7.125	2.748	16.500	2.228
		*F* = 2.607 *p*: 0.79		*F* = 2.325 *p*: 0.103		*F* = 1.183 *p*: 0.311		*F* = 2.045 *p*: 0.135		*F* = 0.912 *p*: 0.405	
Education (degree)	Illiterate	36.250	16.398	18.000	10.132	11.500	4.932	6.750	3.500	25.500	5.259
	Primary school	37.596	12.422	16.649	5.016	13.561	5.650	7.386	4.566	18.508	6.743
	High school	41.964	11.170	18.892	5.363	14.000	4.822	9.071	3.078	17.178	5.347
	Bachelor’s	40.071	10.358	17.857	4.148	14.000	4.057	8.214	3.683	18.000	6.164
		*F* = 0.938 *p*: 0.425		*F* = 1.194 *p*: 0.316		*F* = 0.294 *p*: 0.830		*F* = 1.211 *p*: 0.310		*F* = 2.085 *p*: 0.107	
Income	Income < expense	37.475	14.539	16.450	5.546	13.125	6.152	7.900	4.770	16.850	6.236
	Income = expense	39.960	10.510	18.058	5.151	14.000	4.762	7.902	3.858	19.666	6.544
	Income > expense	40.583	7.856	18.416	4.461	14.000	2.984	8.166	2.405	17.750	5.224
		*F* = 0.587 *p*: 0.558		*F* = 1.276 *p*: 0.284		*F* = 0.346 *p*: 0.708		*F* = 0.022 *p*: 0.978		*F* = 2.308 *p*: 0.105	
Place of residence	City	39.538	12.742	17.092	5.430	13.984	5.398	8.461	4.279	17.984	5.745
	District	36.900	10.896	18.100	4.459	11.800	5.444	7.000	3.554	17.300	5.804
	Village	39.777	10.434	18.166	5.554	14.555	3.501	7.055	3.717	20.833	8.562
		*F* = 0.405 *p*: 0.668		*F* = 465 *p*: 0.630		*F* = 1.716 *p*: 0.185		*F* = 1.500 *p*: 0.228		*F* = 1.772 *p*: 0.175	
Tobacco use	Yes^a^	45.250	10.692	19.687	4.826	15.875	3.649	9.687	3.995	13.750	4.509
	No^b^	38.710	12.191	17.318	5.448	13.724	5.201	7.666	4.185	19.463	5.957
	Quit^c^	34.944	10.485	16.111	4.431	11.444	5.533	7.388	3.483	18.166	7.663
		*F* = 3.383 ***p*: 0.038**		*F* = 2.100 *p*: 0.128		*F* = 3.265 ***p*: 0.042**		*F* = 1.817 *p*: 0.168		*F* = 5.715 ***p*: 0.004**	
Diagnosis	Respiratory system cancer	45.777	14.669	19.888	4.884	15.777	5.651	10.111	4.986	16.333	7.176
	Digestive system cancer	38.491	10.573	16.932	4.645	13.847	4.887	7.711	3.952	18.220	6.236
	Others	38.314	13.246	17.771	6.183	12.800	5.470	7.742	4.002	19.085	6.455
		*F* = 1.569 *p*: 0.213		*F* = 1.329 *p*: 0.269		*F* = 1.285 *p*: 0.281		*F* = 1.420 *p*: 0.247		*F* = 0.692 *p*: 0.503	
Cancer stage	1^a^	42.428	9.966	18.500	5.388	14.071	5.455	9.857	3.880	15.142	6.023
	2^b^	41.527	10.616	18.277	5.218	14.805	4.464	8.444	3.467	17.333	5.918
	3^c^	37.565	12.063	17.130	4.789	13.347	5.398	7.087	3.604	21.347	6.526
	4^d^	35.700	13.658	16.300	5.565	12.333	5.560	7.066	4.891	18.766	6.196
		*F* = 1.831 *p*: 0.147		*F* = 0.991 *p*: 0.401		*F* = 1.317 *p*: 0.273		*F* = 2.072 *p*: 0.109		*F* = 3.461 ***p*: 0.019**	
Do you have comorbidities?	Yes	38.122	11.522	17.390	5.490	13.195	5.011	7.536	3.860	19.365	6.825
	No	39.693	12.310	17.532	5.136	13.967	5.291	8.193	4.226	17.677	6.015
		*t* = 0.422 *p*: 0.517		*t* = 0.295 *p*: 0.894		*t* = 0.198 *p*: 0.461		*t* = 0.016 *p*: 0.426		*t* = 817 *p*: 0.189	

*t*, independent samples *t*-test; *F* = one-way analysis. LSD: a > b > c > d

*Note*: Values in bold indicate statistically significant differences/relationships at the (*p* 0.05) level

According to the correlation analysis, a weak negative relationship was found between age and BES “attribution” scores (*r* = −0.195, *p* = 0.048), while a moderate positive relationship existed between age and ASEX total scores (*r* = 0.381, *p* = 0.000). Similarly, BMI showed a moderate positive correlation with ASEX total scores (*r* = 0.351, *p* = 0.000). Internal scale analysis revealed strong positive correlations between the BES total score and all its subscales (*r* ranging from 0.798 to 0.859, *p* = 0.000). Most importantly, a significant negative correlation was identified between the BES “attribution” subscale and the ASEX total score (*r* = −0.284, *p* = 0.004). This finding indicates that as positive bodily attributions decrease, sexual dysfunction increases among patients. No significant correlation was found between time since diagnosis and any of the scale scores (*p* > 0.05) (Table 3).

**Table 3:** Correlations

Variables	BES total		BES appearance		BES body weight		BES attribution		ASEX total	
	*r*	*p*	*r*	*p*	*r*	*p*	*r*	*p*	*r*	*P*
Age	−0.166	0.094	−0.133	0.179	−0.95	0.339	−0.195*	**0.048**	0.381**	**0.000**
BMI	0.129	0.194	0.115	0.247	0.155	0.118	0.033	0.737	0.351**	**0.000**
Time to diagnosis	−0.055	0.584	−0.77	0.437	−0.009	0.927	−0.049	0.623	−0.040	0.685
ASEX total	−0.111	0.266	−0.002	0.983	−0.030	0.764	−0.284**	**0.004**	1	
BES attribution	0.798**	**0.000**	0.455**	**0.000**	0.597**	**0.000**	1			
BES body weight	0.859**	**0.000**	0.509**	**0.000**	1					
BES appearance	0.814**	**0.000**	1							

*Note*: Values in bold indicate statistically significant differences/relationships at the (*p* 0.05) level

* Correlation is significant at the 0.05 level (2-tailed)

** Correlation is significant at the 0.01 level (2-tailed)

A regression model (enter method) was developed to determine the predictors of body esteem (BES). The model included four variables: weight loss, edema, visible catheter, and tobacco use. The results showed that all four variables were significant determinants of body esteem (*p* < 0.05). The model explained 19.9% of the total variance (*R*^2^ = 0.199), with an adjusted *R*^2^ of 0.167 (*F* = 6.101, *p* = 0.000). Among the predictors, edema (*β* = 0.245) and tobacco use (*β* = −0.231) were the strongest factors influencing body esteem scores. A second regression model was developed to estimate the determinants of sexual life (ASEX). The model included gender, noticeable bodily changes, tobacco use, and cancer stage. Among these, only gender was found to be a significant determinant of sexual life (*β* = −0.321, *p* = 0.001). This model explained 20.0% of the total variance (*R*^2^ = 0.200), with an adjusted *R*^2^ of 0.167 (*F* = 6.123, *p* = 0.000) (Table 4).

**Table 4:** Determinants of scale scores

Scales	Variables						95.0% confidence interval for *B*		Collinearity statistics		Condition index
		*B*	Std. error	Beta	*t*	Sig	Lower bound	Upper bound	Tolerance	VIF	
BES total	(Constant)	5.457	11.548		0.473	0.638	−17.459	28.374			1.000
	Weight loss	4.712	2.205	0.196	2.137	**0.035**	0.337	9.087	0.970	1.031	6.827
	Edema	10.918	4.050	0.245	2.695	**0.008**	2.880	18.955	0.986	1.014	9.520
	Visible catheter	8.165	3.655	0.203	2.234	**0.028**	0.912	15.418	0.990	1.010	15.303
	Tobacco use	−4.796	1.885	−0.231	−2.545	**0.012**	−8.536	−1.056	0.990	1.010	27.494
	*R* = 0.447; *R*^2^ =0.199; adjusted *R*^2^ = 0.167; *F* = 6.101; ***p*** **= 0.000**; Durbin Watson = 1.595										
ASEX total	(Constant)	21.869	4.001		5.466	0.000	13.929	29.808			1.000
	Gender	−4.076	1.177	−0.321	−3.463	**0.001**	−6.411	−1.740	0.948	1.055	5.293
	Noticeable bodily changes	−2.027	1.240	−.155	−1.635	0.105	−4.487	0.433	0.908	1.102	6.866
	Tobacco use	1.954	1.008	0.177	1.939	0.055	−0.046	3.954	0.980	1.020	7.833
	Cancer stage	0.548	0.587	0.090	0.934	0.353	−0.616	1.713	0.887	1.128	16.969
	*R* = 0.447; *R*^2^ =0.200; adjusted *R*^2^ = 0.167; *F* = 6.123; ***p*** **= 0.000**; Durbin Watson = 1.644										

*Note*: Values in bold indicate statistically significant differences/relationships at the (*p* 0.05) level

## Discussion

Research findings indicate that body esteem scores among Turkish cancer patients are below average, predominantly predicted by physical and clinical variables. Recent studies confirm that patients undergoing active treatment protocols report lower body esteem compared to those in the survivorship phase, emphasizing the acute impact of clinical interventions [29]. Regression analysis results showed that weight loss, the presence of edema, visible catheter use, and smoking status are significant predictors of the total variance in body esteem. The finding that weight loss experienced during the illness process lowers body esteem is consistent with common findings in the literature. Involuntary weight loss triggered by cancer cachexia and treatment side effects is considered a primary source of stress for patients[4]. Recent research highlights that weight-related changes are among the top three distress factors for oncology patients, significantly driving social isolation [30]. However, one of the most striking findings of the study is the detrimental impact of the presence of edema on body esteem. Edema is defined as a complication that disrupts body symmetry and impairs the patient’s aesthetic perception [31]. Another important physical marker is the presence of visible catheters outside the body. Invasive treatments and visible clinical signs, such as catheters, negatively alter physical appearance and reduce body esteem [3]. Current research suggests that “body integrity” is significantly compromised by medical devices, which patients often perceive as a permanent mark of their illness [32].

On the other hand, our research findings show that sexual dysfunction levels among Turkish cancer patients are quite common, with mean scores consistently exceeding clinical cut-off points. Gender emerged as the most decisive factor in sexual function. Contemporary studies in Middle Eastern and Mediterranean cultures (2022–2024) continue to highlight gender-based disparities, noting that cultural silence surrounding female sexuality exacerbates dysfunction [33–36]. The more pronounced levels of sexual dysfunction reported by female patients compared to males can be explained by traditional Turkish social norms, where female sexuality is more restricted and experienced within the boundaries of “mahremiyet” (privacy) [10, 11].

The progressive nature of the disease also plays a decisive role in sexual health. In our study, it was found that patients in the advanced stages of cancer had worse sexual functions. This finding aligns with studies indicating that symptom burden increases and sexual problems deepen as the disease stage progresses [37]. Evidence suggests that healthcare providers rarely initiate discussions on sexual health with cancer patients, effectively marginalizing patients’ sexual identity within the clinical encounter—a pattern particularly pronounced in culturally conservative contexts [34]. Health professionals should move beyond survival-oriented care and develop holistic support strategies that include pre-treatment information regarding bodily changes and sexual dysfunctions.

### Conclusion and recommendations

This study concludes that cancer patients experience significant challenges characterized by low body esteem and widespread sexual dysfunction. The findings show that body esteem is primarily determined by physical and clinical factors such as cancer-related weight loss, edema, and the presence of visible catheters, as well as lifestyle factors such as tobacco use. Collectively, these variables explain a significant portion of the variance in patients’ self-perception. Furthermore, sexual life is significantly affected by gender, BMI, and cancer stage; women, patients in advanced stages, the elderly, and individuals with high BMI are at higher risk for sexual health problems. Although the total scores for body esteem and sexual life did not show a direct causal relationship in the regression model, the significant correlation between sexual life and the “attribution” subscale of body esteem suggests that these two areas are psychologically intertwined and that the value a patient places on their body acts as a bridge to sexual well-being.

#### Recommendations for clinical practice and research

Based on the findings of this study, several critical recommendations are offered to enhance holistic oncology care. First, healthcare professionals, particularly oncology nurses, must extend their focus beyond survival-focused care by routinely assessing patients for body image distress and sexual dysfunction using validated measurement tools. Furthermore, to mitigate psychological distress through preparedness, patients should be proactively informed about potential bodily changes, such as edema and weight fluctuations, as well as the long-term impact of medical devices like catheters.

#### Tailored and inclusive interventions

In addition to routine assessments, gender-sensitive counseling and specialized sexual health interventions should be actively integrated into oncology rehabilitation programs, with particular emphasis on high-risk female patients who face greater cultural and social vulnerabilities. Moreover, BMI management and smoking cessation support should not be viewed merely as physical health targets; instead, they should be utilized as strategic psychosocial interventions aimed at improving body esteem and sexual well-being. Finally, to effectively manage relational and social anxieties, spouses or partners should be systematically included in the treatment and support process, ensuring a more comprehensive support system for the patient.

## Limitations of the study

*Study population and diagnostic heterogeneity*: This study’s primary limitation concerns the diagnostic distribution of the sample. To control for direct anatomical and mechanical disruptions to sexual function, patients with reproductive system cancers (e.g., prostate, cervical, or ovarian cancers) were excluded. Consequently, the sample was predominantly composed of patients with digestive system cancers (57.3%), while those with respiratory system cancers (8.7%) and other non-reproductive malignancies (34.0%) were less represented. This uneven distribution and the exclusion of genital cancers limit the generalizability of the findings to the broader oncological population.

*Cultural sensitivity and reporting bias*: The research was conducted in a society where more than 90% of the population identifies as Muslim. In such contexts, sexual health remains a sensitive and often taboo subject, which may have led to social desirability bias. Consequently, some participants may have underreported their sexual dysfunction or felt uncomfortable providing precise answers to the ASEX items.

*Scale reliability*: A secondary limitation concerns the internal consistency of the scales used. The Cronbach’s alpha values for certain subscales were found to be lower than the ideal threshold. This relatively low reliability may be attributed to the clinical distress of the participants or cultural nuances in how the questions were perceived, potentially affecting the precision of measurement for those specific domains.

*Generalizability:* The data were collected from a single healthcare institution. Therefore, the results reflect the experiences of a specific patient group and may not be fully generalizable to the entire population of cancer patients in Turkey or other diverse cultural settings.

### Clinical implications

The findings of this study have significant implications for oncological nursing and supportive care practices. Oncology nurses are in a strategic position to routinely evaluate patients for body esteem issues and sexual dysfunction. Based on our results, it is highly recommended that healthcare providers involve spouses or partners in the treatment process to address relational and social concerns effectively. Furthermore, a multidisciplinary approach is essential; oncology nurses should actively collaborate with and, when necessary, refer patients to psychiatric nurses or sexual health counselors for specialized, targeted interventions. Utilizing these approaches will enhance nurses’ awareness regarding these two often-neglected aspects of cancer care, thereby facilitating the planning and implementation of holistic treatment strategies.

### Conclusions

In conclusion, this study underscores that physical and clinical variables heavily influence body esteem, while gender and cultural norms remain decisive factors in the sexual function of Turkish oncology patients. To build upon these findings, future research should be designed to include more diverse patient populations, encompassing different cancer types and participants from various religious and cultural backgrounds. Additionally, further longitudinal studies are needed to develop and test specific interventions—such as psychoeducational programs or structured support groups—aimed at mitigating these challenges and improving the overall quality of life for cancer survivors.

## Data Availability

No datasets were generated or analysed during the current study.
